# Parental Education Moderates the Relation between Physical Activity, Dietary Patterns and Atopic Diseases in Adolescents

**DOI:** 10.3390/children9050686

**Published:** 2022-05-09

**Authors:** George Antonogeorgos, Demosthenes B. Panagiotakos, Kostas N. Priftis, Evangelia Liakou, Alexandra Koutsokera, Pavlos Drakontaeidis, Marina Thanasia, Maria Mandrapylia, Dafni Moriki, Philippa Ellwood, Luis García-Marcos, Konstantinos Douros

**Affiliations:** 1 Allergology and Pulmonology Unit, 3rd Paediatric Department, National and Kapodistrian University of Athens, 12462 Athens, Greece; kpriftis@otenet.gr (K.N.P.); drliakouevangelia@yahoo.gr (E.L.); alexandra.koutsokera@gmail.com (A.K.); pauldrakos@hotmail.com (P.D.); marinarod9422@gmail.com (M.T.); marmand24@outlook.com (M.M.); dafnimoriki@yahoo.gr (D.M.); costasdouros@gmail.com (K.D.); 2 Department of Nutrition and Dietetics, School of Health Sciences and Education, Harokopio University, 17676 Athens, Greece; dbpanag@hua.gr; 3 Department of Pediatrics: Child and Youth Health, Faculty of Medical and Health Sciences, University of Auckland, Auckland 1023, New Zealand; p.ellwood@auckland.ac.nz; 4 Pediatric Allergy and Pulmonology Units, Virgen de la Arrixaca University Children’s Hospital, University of Murcia, Network of Asthma and Adverse and Allergic Reactions (ARADyAL) and Biomedical Research Institute of Murcia, IMIB-Arrixaca, 30394 Murcia, Spain; lgmarcos@um.es

**Keywords:** parental, education, diet, physical, activity, allergy, adolescents, prevalence

## Abstract

Background: Atopic diseases, particularly asthma, eczema, and rhinitis, are among the most common chronic diseases in childhood, with several factors implicated in their pathogenesis. Our study examined the role of parental education in the association between diet, physical activity, and atopy in adolescents. Methods: 1934 adolescents (47.5% boys) aged 13–14 years old reported information about their diet and physical activity and their parents reported their highest educational level. The moderating role of parental education level (primary/secondary vs. tertiary) in the relation between lifestyle patterns and atopic diseases was examined with logistic regression analyses. Results: High consumption of dairy products was inversely associated to adolescents’ asthma and rhinitis symptoms overall, but this relation was almost 50% stronger for the adolescents with high parental education level background. The same pattern of reduction of the odds was noticed also regarding the association among the high intake of fruits, vegetables, pulses, with all three atopic diseases and the adherence to a physically active lifestyle only with current asthma and eczema (all *p* < 0.05). Conclusion: Adolescents who are physically active and consume a higher intake of fruits, vegetables, and pulses and a lower intake of fast-food and sweets, and their parents/guardians having higher education, are less likely to have any current symptoms of asthma, eczema, and rhinitis than the ones who have low educated parents.

## 1. Introduction

Atopic diseases, particularly asthma, eczema, and rhinitis, are common chronic diseases in childhood [1]. Pediatric asthma is a major public health problem and it has serious consequences for any asthmatic child and his/her family, affecting their quality of life and school attendance and work, correspondingly. The worldwide rates of pediatric asthma vary between 10–15% [2]. Similarly, eczema prevalence varies from 15 to 20%, whereas allergic rhinitis rates rise up to 40%, making the broad range of atopic diseases a serious threat for public health [3,4].

Several factors have been implicated in the etiology of asthma and its exacerbations: genetic, lifestyle, and environmental, and among the most critical are physical activity and dietary habits. Emerging evidence about the contribution of physical activity in the etiology of atopy suggests that children and adolescents with low levels of physical activity have higher risk of developing asthma and current asthma symptoms or wheeze; however, there is also evidence about a vice-versa relationship, since children with asthma and eczema are prone to sedentary behaviors [5,6]. Health, mainly plant-based eating patterns, such as the Mediterranean diet, have been associated with lower risk of asthma and rhinitis in several studies and meta-analyses [7,8,9], whereas others have reported that diets rich in legumes and potatoes were associated with increased risk of allergic rhinitis in primary school children [10].

Socioeconomic status (SES) is a critical determinant of health, in both adults and children, worldwide. Despite the advances that have been established in social healthcare, and the equity in healthcare access, there is a plethora of evidence associating low SES and other poverty-related exposures with asthma onset and poor asthma outcomes, such as low pulmonary function and frequent exacerbations [11,12,13]. However, opposing associations have also been observed; in a British birth cohort study, children coming from high SES families had higher likelihood of having atopic dermatitis. All these findings contribute to the difficulty in making robust conclusions about the role of SES on asthma and other atopic disease development [14]. Parental education level is often used as an indicator for family SES but also plays a critical role in atopic diseases per se. Children with low education parents had an increased likelihood of inpatient and outpatient asthma diagnosis as well as poorer asthma treatment but lower risk of inhalant allergy and itchy rash compared to families where at least one parent had a college degree [15,16].

To the best of our knowledge, no assessment of the possible moderating role of the parental education in the relationship between lifestyle patterns and the prevalence of atopic diseases in childhood has ever been examined in the relevant literature. The Global Asthma Network study (GAN) was established and designed to assess the worldwide symptom prevalence of asthma, eczema, and rhinitis and to identify several environmental factors associated with them in children aged 6–7 and 13–14 years old [17]. In order to further expand the GAN research question, we hypothesized in this study that parental education moderates the associations between dietary and lifestyle patterns with asthma, eczema, and allergic rhinitis symptoms in adolescents aged 13–14 years old living in an urban environment in Greece.

## 2. Materials and Methods

This is a cross-sectional, observational study, part of GAN Phase I study, which is an international project aimed to monitor the worldwide prevalence, severity, management, and risk factors of asthma and other atopic diseases such as allergic rhinitis and eczema, in two-time periods of childhood, i.e., 6–7 and 13–14 years old [2].

The Greek part of the GAN Phase I included adolescents aged 13–14 years and their parents/guardians and followed the same protocol as the GAN Phase I study. The sampling took place in the greater metropolitan area of Athens, Greece, from February to March 2020, in a high-schools’ setting; 20 high schools were selected by convenience sampling, from a list that was provided by the Secondary Education Office in Athens. All children in the 1st and 2nd grades of each high-school were asked to participate. Schools for children with special educational needs or disabilities were excluded. Table 1 presents the characteristics of the study sample. The study was approved by the ethics committee of the National and Kapodistrian University of Athens (decision number: 214/13-12-19). For the accomplishment of the study, permission was issued by the Ministry of Educational Affairs (decision number: 10053/24-01-2020).

The GAN study included two standardized questionnaires with several questions assessing the occurrence of atopic symptoms and various lifestyle characteristics: one that was completed by the adolescents during schooltime and one intended for parents/guardians to complete at home (adult questionnaire). Specifically, current asthma was defined as the positive answer to the question “Have you had wheezing or whistling in the chest in the past 12 months?” Similarly, current rhinitis was defined as the positive answer to the question “In the past 12 months, have you had a problem with sneezing or a runny or blocked nose when you did not have a cold or the flu?” and current eczema as the positive answer to the question “Have you had this itchy rash at any time in the past 12 months?”. Moreover, adolescents were asked if they had any siblings, and if so, how many. Adolescents’ parents or guardians were also asked to report if they had a history of atopic diseases (asthma, eczema, or allergic rhinitis) if there was visible moisture or mold spots on the walls or ceiling of their homes and whether they smoke. The participating parental/guardian educational level was recorded in three categories (primary (compulsory education/9 years), secondary (non-compulsory/3 years), or tertiary (university/college/post-graduate studies)). Due to the small number of parents who had primary education (*n* = 24), we merged this level with the next one (secondary education), creating a dichotomous variable (i.e., parental primary/secondary educational level vs. tertiary).

The GAN questionnaire included a validated 22-item Food Frequency Questionnaire (FFQ) assessing the past 12-month consumption frequency of 22 food groups or food items [18]. More specifically, adolescents answered 22 questions related to the consumption frequency of 10 food groups, namely, meat, sea-food, including fish, fruits, cooked vegetables (green and root), raw vegetables (green and raw), pulses (peas, beans, lentils), cereals, dairy (cheese and yoghurt), sugar (including lollies/candies/sweets), fast food (excluding burgers), and fizzy or soft drinks and 12 food items (bread, pasta, rice, margarine, butter, olive oil, milk, eggs, nuts, potatoes, fast-food (burgers), choosing one of the three following options in the past 12 months: never or only occasionally, once or twice per week, and most of all days. The consumption of meat, seafood, fruits, cooked and raw vegetables, pulses, cereals, bread, pasta and rice, olive oil, milk and dairy products, nuts, fast-food, sweets and candies, and soft drinks were further recoded into two categories, in order to classify adolescents into high (most or all of the days in the past 12 months) vs. low (never up to twice per week in the past 12 months) consumption per week. Regarding physical activity of the participating adolescents, it was assessed through three detailed questions: (a) the number of occasions per week engaging in vigorous physical activity up to the point of breathing hard with the following possible answers: never or occasionally, once or twice per week, and three or more times per week and (b) the number of hours a day watching television/film or videos and the number of hours per day engaging in computer and internet activities (gaming, chatting, Facebook, YouTube, etc.) with the following possible answers: less than one hour, over one and up to three hours, over three and up to five hours, and more than five hours per day. Likewise, these questions were further recoded and adolescents were classified in two categories regarding their level of vigorous physical activity (high vs. low if they were engaging in vigorous physical activity until it is difficult to breathe for at least three times per week vs. up to twice per week) and daily sedentary activity due to TV watching or computer and internet activities (increased vs. de-creased if adolescents were engaging for more than three hours per day or more vs. up to three hours daily).

Furthermore, participants, according to their answers in the physical activity and dietary assessment questions, were categorized into five lifestyle patterns. Adolescents who were classified as having a high physical activity level per week and low TV, computer, and internet daily engagement were characterized as adherent to an active lifestyle with physical activity and as high consumers of the following food groups: (a) fruits, vegetables, and pulses, (b) carbohydrates (cereal, bread, pasta, rice), (c) all dairy products (milk, cheese, yogurt), if they were consuming these foods most days per week. Moreover, they were also categorized as low consumers of unhealthy food (fast-food, sweets, lollies, and soft drinks) if they were consuming any of the aforementioned food groups up to twice a week.

Continuous variables are presented as mean and standard deviation (SD), and categorical variables are presented as absolute and relative frequencies. Pearson Chi-square and Fisher’s exact test—when appropriate—as well as Student’s *t*-test were applied to examine for univariate differences among the several qualitative and quantitative physical activity and dietary characteristics of adolescents and current asthma, allergic rhinitis, and eczema symptoms status, respectively. Moreover, univariate and multivariate logistic regression models (adjusted for several well-known confounders based on the related literature), crude and stratified by the parental education level (primary/secondary vs. tertiary), which was considered as a proxy of SES, were applied to estimate the adolescents’ odds ratios (OR) and the corresponding 95% Confidence Intervals (95% CI) of allergic outcomes based on the defined five lifestyle factors. The log-likelihood ratio test was used to evaluate interactions between the lifestyle patterns and parental education level by comparing the models with and without the cross-sectional product term of these variables. All reported probability values (*p*-values) were based on two-sided tests and compared to a significant level of 5%. STATA 14 software was used for all the statistical calculations (STATA Corp., College Station, TX, USA).

## 3. Results

From the total study sample of 1934 adolescents, 6.9% reported that they had symptoms of current asthma (at least one episode of wheezing or whistling in the chest in the past 12 months), 25.3% that they had symptoms of current rhinitis (at least one episode of sneezing or runny nose without cold symptoms in the past 12 months), and 8.9% they had symptoms of current eczema (at least the appearance of an itchy rash in the past 12 months). More information about the characteristics of the study sample can be found elsewhere [19]. Results from the univariate analysis between sex and several anthropometric, parental, indoor home environment, and the lifestyle patterns of the adolescents with the atopic study outcomes are presented in Table 2.

To further explore the associations between the dietary and physical activity patterns with the current asthma, allergic rhinitis, and eczema symptoms, univariate (with the lifestyle factors as the only independent variable) and multivariate logistic regression analyses were applied, adjusted for all the remaining lifestyle factors: sex, obesity status, parental atopic history, pet ownership, parental smoking, having an older sibling, indoor exposure to dampness and/or mold. Moreover, the interaction term between parental education and lifestyle patterns was also examined. In Table 3, the results of the univariate and multivariate logistic regression models with the *p*-values of the interaction terms between parental education and the various lifestyle patterns are presented. Asthma symptoms in the past 12 months were inversely associated with high consumption of fruits, vegetables, and pulses and dairy products per week (OR: 0.27, 95% CI: 0.12–0.60 and 0.61, 0.42–0.89, respectively). Regarding allergic rhinitis symptoms, similar significant inverse associations were observed with increased consumption of fruits, vegetables, pulses, and dairy products and with low consumption of unhealthy foods (i.e., fast-food, sweets, lollies, and soft drinks) (OR: 0.27, 95% CI: 0.50–0.96 and 0.73, 0.59–0.90, correspondingly). Current eczema was inversely related with a physically active lifestyle and with high consumption of fruits, vegetables, and pulses (OR: 0.59, 95% CI: 0.38–0.91 and 0.46, 0.25–0.83, respectively). In addition, significant interactions were observed between parental education level and adherence to an active lifestyle pattern on current asthma and eczema symptoms development; similarly, between parental education level and consumption of fruits, vegetables, and pulses and dairy products, as well as low consumption of unhealthy foods on all atopic outcomes (all *p* < 0.05).

In Table 4, the results of the analyses for the relation of each lifestyle factor on current asthma, rhinitis, and eczema symptoms, stratified according to each level of parental education (primary/secondary vs. tertiary), are presented. High consumption of dairy products was inversely related to adolescents’ asthma and rhinitis symptoms in both strata, but this relation was almost 50% stronger for the adolescents with high parental education level background. The same pattern of reduction of the odds between adolescents living in high and low parental education level families was noticed also regarding the association among the high consumption of fruits, vegetables, pulses with all atopic diseases and the adherence to a physically active lifestyle only with current asthma and eczema (all *p* < 0.05).

## 4. Discussion

This study of adolescents living in an urban setting in Greece demonstrated the important moderating role of parental education and, by extension, family SES, in the relationship between lifestyle patterns with atopic diseases. It was observed that parental education, and particularly low parental education, plays a critical moderating role in the relationships between adolescents’ dietary habits, engagement in physical activities and presence of atopic diseases. Most interesting is the finding that low parental education suppresses the beneficial association between adolescents’ adherence to a healthy eating consumption pattern and an active lifestyle with atopic diseases. Thus, our study provides new insights that could benefit public health policy makers in planning tailor-made and sustainable preventive strategies against atopic diseases in childhood by targeting appropriately specific groups according to the parental education level. Furthermore, any health care worker involved in the management of atopic adolescents should be more vigorous in his/her advice related to lifestyle changes for adolescents originated from low parental education level and subsequently low family SES, to additionally improve their care.

The important role of the family’s SES as a risk factor in the prevalence of atopic diseases in childhood atopy has been noted in the relevant literature, but with conflicting results. In the study by Simon et al., a negative association was found between the prevalence of asthma and home income in all ethnic and racial groups [20]. Family low education level was also marked as the most important factor in the relation between race and asthma in the study of 499 families living in an urban environment in the United States [21]. The same finding is also reported in a children’s sample from Lanzhou, China, and from a national representative sample of Swedish children [22,23]. Moreover, Kojiima et al. reported that children living in households with a low annual income had higher prevalence of doctor-diagnosed asthma and eczema [24]. However, there are also numerous studies reporting the opposite relationship among SES and allergic diseases. A multicenter study in seven cities in China reported that higher SES was positively associated with asthma and rhinitis among preschool children [25]. This finding is also observed in a cohort study of 3979 South African adolescents [26]. Allergic sensitivity, demonstrated as positive skin prick tests in several aeroallergens was higher in high-SES children living in an urban area of Indonesia [27]. Similar results are also found elsewhere [28,29]. Our study not only advocate the important role of of parental education level and, thus, SES in the prevalence of atopic diseases but further expands it as a modifier of the association between lifestyle patterns and atopic diseases. Thus, knowledge of parents’ educational status could not only help clinicians and public health workers to identify more vulnerable children populations to atopic diseases, but also provides them with an early assumption about the effect of their lifestyle interventions.

The beneficial role of high family SES status in the association between the healthy dietary patterns and atopic diseases in adolescence could be related to the richest diet quality that adolescents living in high SES environment exhibit. There are numerous studies documenting the association between lower SES family status and bad diet quality in children. In a recent study by Gangrade et al., in the context of the National Health and Nutrition Examination Survey (NHANES) in the United States, adolescents from lower SES status families had lower probability of consuming milk and dairy products and fruits as snacks and higher probability of consuming more added sugar and less fibers than the ones living in high SES level families [30]. Moreover, lower parental education was associated with lesser likelihood of a healthy dietary eating pattern in adolescents from a southeastern region of the United States [31]. Most interesting is the evidence reported by Michels et al., who revealed that up to 64% of the mediating effect of healthy eating determinants between SES status and diet quality could be attributed to psychosocial determinants such as parental influence and awareness about healthy eating patterns [32]. Thus, the observed positive influence of the healthy dietary patterns in the prevalence of the atopic diseases in our study could be attributed to the higher level of adoption of healthy eating patterns among adolescents living in high SES families. Nevertheless, we noted that, although to a lesser degree, the influence of a dietary pattern rich in fruits, vegetables, pulses, and dairy products and poor in fast-food and sweets remains beneficial in atopic adolescents with parents of low educational level and this specific group of adolescents should be carefully targeted and further encouraged to adopt even healthier eating patterns.

Another interesting finding of our study is the almost half lesser inverse association between adherence to a physically active lifestyle pattern and current asthma and eczema symptoms for adolescents living with highly educated parents. This could be attributed to the positive association between physical activity and high family SES in children. There are a number of studies supporting this. Drenowatz et al. divided a sample of children aged 8–11 years old according to their household annual income and found a direct relationship between steps/day and family SES. Children in families with the higher total annual income walked significantly more steps/day than the lowest ones [33]. Home environment of adolescents living in low SES families provides more opportunities for sedentary activities such as greater access to screens (television, videogames) in their bedrooms but lower access to portable exercise equipment such as bikes, jump ropes, etc. [34]. Moreover, children’s screen time has been inversely related with SES while more higher SES children participated in sports activities, as demonstrated in the study by Fairclough et al. [35]. Finally, low-SES families tend to reside in low-SES neighborhoods and there is evidence that the neighborhood-built environment is related to several physical activity behaviors [36].

High family SES advocates the adoption of more active and healthier dietary lifestyle patterns and these patterns have been found to positively affect the inflammatory pathophysiological mechanisms involved in the pathogenesis of atopic diseases by down-regulating numerus inflammatory mediators. It is reported that exercising on a regular basis can induce significant reduction in proinflammatory cytokines, thus, reducing the levels of allergic inflammation [37]. Moreover, physical fitness can reduce inflammation in the airways by reducing expression of Th2 cytokines, mainly IL-4, IL-5 and IL-13 [38]. The association of low SES with poor asthma outcomes in childhood could also be facilitated through the higher levels of chronic stress they suffer, and which is associated with increased production of the cytokines IL-5 and IL-13 as well as with higher eosinophil counts in blood samples, as it was shown in the study by Chen et al. [39]. Furthermore, the effect of low SES during early childhood can affect immune programming, resulting in phenotypes prone to inflammation in mid-life adults [40]. Regarding the protective moderating role of high parental education level in the inverse association between fruits, vegetables, and pulses consumption and atopic diseases, this could be attributed to the improved diet quality and the healthier dietary patterns that high SES families tend to adhere to. Results from the multi-national ISCOLE study evident that lower parental education levels were related with higher intake of unhealthy foods and lower intake of healthy ones in 9–11-year-old children living in 12 countries [41]. Higher adherence to healthier dietary patterns leads to higher antioxidant intake and there is a plethora of evidence about the beneficial role of a diet rich in antioxidants in atopic diseases. In a Swedish birth-cohort study of 2506 participants, it was found that a higher intake of fruits and total antioxidants at age 8 was associated with lower odds of prevalent and incidence asthma at adolescence and up to 24 years [42,43]^,^. Moreover, specific antioxidants such as b-carotene and magnesium have been inversely correlated with the prevalence of rhinitis and atopic sensitization [44]. Finally, in another birth cohort study from the United Kingdom, dietary antioxidant of children living in Manchester at age 5 was associated with allergic sensitization at age 8 [45].

The study has a cross-sectional design, and it suffers the inherited limitations of this type of epidemiological studies, however, every effort was made not to over-interpret the study results. However, every effort was made not to over-interpret the study results. The assessment of atopic diseases included only self-reported symptoms during the last 12 months and could be prone to recall and reporting bias. However, the use of a validated questionnaire and the worldwide application of it reassures the minimization of it and allows the comparability of our results between studies [18]. Moreover, the moderating association of SES could be attributed to the worse home-environment conditions that adolescents of low-SES families might be experiencing, but our results have taken this into account by adding as a confounder in all our multivariate models the presence of visible dampness and/or mold spots indoors. Finally, obesity could be a factor associated with the modulatory role of SES in the relation between atopy and diet, but also, we have taken this effect into account by adding it also as a confounder in all our multivariate models.

## 5. Conclusions

Our study highlights the moderating role of parental education level, and subsequently family SES, on the association between healthy dietary, activity lifestyle patterns and atopy. Thus, adolescents who are physically active and consume a higher intake of fruits, vegetables, and pulses and a lower intake of fast-food and sweets and whose parents/guardians have higher education are less likely to have any current symptoms of asthma, eczema, and rhinitis. Public health policymakers and clinicians implicated in the care of atopic adolescents should be aware of the importance of parental education during the development of preventive lifestyle interventions or their clinical advice, correspondingly, in order to reduce the burden of these major chronic diseases in adolescence.

## Figures and Tables

**Table 1:** Characteristics of the Greek GAN study sample.

Total sample (child–parent/guardian pairs, *n*)	2560
Study sample (child–parent/guardian pairs, *n*)	1934
Adolescent boys (*n*, (%))	921, 47.5
Adolescents’ age (mean, (SD *) years)	12.7 (0.6)
Adolescents’ fathers (*n*, (%))	492, 25.4
Paternal age (mean, (SD) years)	49.1 (5.5)
Maternal age (mean, (SD) years)	45.4 (4.8)

* SD: Standard Deviation.

**Table 2:** Characteristics of the study adolescents according to their atopic diseases’ history status and parental educational level (*n* = 1934).

	Adolescent’s Asthma Symptoms in the Past 12 Months (Current Asthma)		Adolescent’s Allergic Rhinitis Symptoms in the Past 12 Months (Current Rhinitis)		Adolescent’s Allergic Rash Symptoms in the Past 12 Months (Current eczema)	
**Parental Education level: Primary/Secondary (** ***n*** **= 654)**						
	**No**	**Yes**	**No**	**Yes**	**No**	**Yes**
*n*	597	57	470	184	601	53
Boys (*n*, %)	266 (44.7)	24 (42.1)	203 (43.4)	87 (47.3)	271 (45.2)	19 (35.8)
Pet ownership (Yes, *n*, %)	184 (31.0)	21 (36.8)	146 (31.3)	59 (32.1)	185 (30.9)	20 (37.7)
Having an older sibling (Yes, *n*, %)	281 (47.3)	25 (43.9)	223 (47.8)	83 (45.1)	277 (46.3)	29 (54.7)
Parental atopic history (Yes, *n*, %)	41 (6.9)	14 (7.0)	**26 (5.5)**	19 (10.3) *	40 (6.7)	25 (9.4)
Parental ever smoking (Yes, *n*, %)	370 (62.1)	38 (66.7)	280 (59.7)	128 (69.6)	369 (61.5)	39 (73.6)
Overweight/Obese adolescents (Yes, *n*, %)	218 (36.6)	27 (47.4)	16 3 (34.8)	82 (44.6)	220 (36.7)	25 (47.2)
Indoor exposure to dampness and/or mold (Yes, *n*, %)	131 (21.9)	10 (17.5)	**93 (19.8)**	**48 (26.1)**	125 (20.8)	16 (30.2)
Adherence to an active physical activity lifestyle ^†^	74 (12.4)	14 (3.5)	48 (10.2)	28 (15.2)	74 (12.3)	28 (11.2)
High consumption of fruits, vegetables, and pulses per week (most or all days)	**175 (29.3)**	**12 (3.5)**	126 (26.8)	51 (27.7)	**169 (28.1)**	**18 (15.1)**
High consumption of carbohydrates (bread, pasta, and rice) per week (most or all days)	36 (6.0)	14 (0.5)	25 (5.3)	13 (7.1)	33 (5.5)	15 (9.4)
High consumption of dairy (milk, yogurt, and cheese) per week (most or all days)	**284 (46.6)**	**19 (33.3)**	221 (47.0)	82 (44.6)	275 (45.8)	28 (52.8)
Low consumption of unhealthy foods (fast-food, sweets, and soft drinks) per week (up to twice)	**348 (58.3)**	**23 (40.4)**	276 (58.7)	95 (51.6)	**348 (57.9)**	**23 (43.4)**
**Parental Education level: Tertiary (*n* = 1280)**						
*n*	1205	75	470	184	1161	119
Boys (*n*, %)	590 (49)	40 (53.3)	475 (48.8)	155 (50.7)	578 (49.8)	52 (43.7)
Pet ownership (Yes, *n*, %)	335 (27.8)	23 (30.7)	269 (27.6)	89 (29.1)	318 (27.4)	40 (33.6)
Having an older sibling (Yes, *n*, %)	490 (40.7)	33 (44.0)	405 (41.6)	118 (38.6)	464(40.0)	59 (49.6)
Parental atopic history (Yes, *n*, %)	97 (8.0)	13 (17.3)	26 (5.5)	19 (10.3)	101 (8.7)	9 (7.6)
Parental ever smoking (Yes, *n*, %)	631 (52.3)	43 (57.3)	**74 (7.6)**	**36 (11.8)**	608 (52.3)	66 (55.5)
Overweight/Obese adolescents (Yes, *n*, %)	351 (29.1)	27 (36.0)	504 (51.6)	170 (55.6^)^	346 (29.8)	32 (26.9)
Indoor exposure to dampness and/or mold (Yes, *n*, %)	**293 (24.3)**	**26 (34.7)**	238 (24.4)	81 (26.5)	**277 (23.8)**	**42 (35.3)**
Adherence to an active physical activity lifestyle	353 (29.2)	18 (24.0)	281 (28.8)	90 (29.4)	346 (29.8)	25 (21.0)
High consumption of fruits, vegetables, and pulses per week (most or all days)	98 (8,1)	15 (6.7)	**97 (9.9)**	**16 (2.0)**	98 (8.4)	12 (4.2)
High consumption of carbohydrates (bread, pasta, and rice) per week (most or all days)	48 (4.0)	14 (8.0)	40 (4.1)	14 (4.6)	48 (4.1)	18 (5.0)
High consumption of dairy (milk, yogurt, and cheese) per week (most or all days)	**631 (52.3)**	**30 (40.0)**	**528 (54.1)**	**133 (43.5)**	601 (51.7)	60 (50.4)
Low consumption of unhealthy foods (fast-food, sweets, and soft drinks) per week (up to twice)	697 (57.7)	41 (54.7)	**590 (60.5)**	**148 (48.4)**	675 (58.0)	63 (52.9)

* Bold numbers denote *p* < 0.05 ^†^ Engaging in vigorous physical activity for >3 h/day plus watching TV and engaging in computer and internet activities for less than 3 h.

**Table 3:** Results from the multiple logistic regression analysis assessing the association between history of an atopic disease (asthma, rhinitis, eczema) in the past 12 months and adolescents’ lifestyle factors, adjusted for several confounders with the corresponding *p*-value derived from the log-likelihood ratio test of the multivariate model with and without the interaction term between each lifestyle pattern with parental educational level (*n* = 1934).

	Adolescent’s Asthma Symptoms in the Past 12 Months (Current Asthma)		*p* for Interaction ^$^	Adolescent’s Allergic Rhinitis Symptoms in the Past 12 Months (Current Rhinitis)		*p* for Interaction	Adolescent’s Allergic Rash Symptoms in the Past 12 Months (Current Eczema)		*p* for Interaction
**Lifestyle patterns**	Crude OR (95% CI) ^§^	Adjusted ^†^ OR (95% CI)		Crude OR (95% CI)	Adjusted OR (95% CI)		Crude OR (95% CI)	Adjusted OR (95% CI)	
Adherence to an active physical activity lifestyle every week ^‡^ (Yes vs. No)	0.59 * **(0.36–0.96)**	0.67 (0.40–1.10)	0.012	**0.61** **(0.40–0.94)**	1.21 (0.94–1.55)	0.58	1.07 (0.84–1.37)	**0.59** **(0.38–0.91**)	0.007
High consumption (most or all days) of fruits, vegetables, and pulses per week (Yes vs. No)	**0.27** **(0.11–0.56)**	**0.27** **(0.12–0.60)**	<0.001	**0.45** **(0.25–0.82)**	**0.29** **(0.35–0.82)**	<0.001	**0.73** **(0.53–0.99)**	**0.46** **(0.25–0.83)**	0.029
High consumption (most or all days) of starchy products (cereal, bread, pasta, and rice) per week (Yes vs. No)	1.25 (0.59–2.66)	1.21 (0.57–2.60)	0.44	1.36 (0.71–2.62)	1.22 (0.76–1.95)	0.68	1.2 (0.76–1.91)	1.26 (0.63–2.38)	0.74
High consumption (most or all days) of dairy (milk, yogurt, and cheese) per week (Yes vs. No)	**0.59** **(0.41–0.85)**	**0.61** **(0.42–0.89)**	0.007	1.05 (0.77–1.44)	**0.73** **(0.59–0.90)**	0.032	0.73 (0.59–0.90)	1.11 (0.81–1.52)	0.48
Low consumption (up to twice) of unhealthy foods (fast-food, sweets, lollies, and soft drinks) per week (Yes vs. No)	**0.69** **(0.49–0.98)**	0.72 (0.51–1.04)	0.015	**0.65** **(0.53–0.80)**	**0.71** **(0.53–0.92)**	0.021	0.61 (0.51–0.79)	0.76 (0.55–1.05)	0.79

^†^ Adjusted for sex, obesity status, parental atopic history, pet ownership, parental smoking, having an older sibling, exposure to indoor dampness and/or mold ^‡^ engaging in vigorous physical activity for >3 h/day plus watching TV and engaging in computer and internet activities for less than 3 h per day (*n*, %) ^§^ OR (95% CI): Odds Ratio (95% Confidence Interval) * Bold numbers denote *p* < 0.05 ^$^ interaction among each lifestyle factor with parental education level.

**Table 4:** Results from the multiple logistic regression analysis assessing the association between history of an atopic disease (asthma, rhinitis, eczema) in the past 12 months and adolescents’ lifestyle factors, adjusted for several confounders and all the remaining lifestyle factors according to their parental educational level.

	Adolescent’s Asthma Symptoms in the Past 12 Months (Current Asthma)		Adolescent’s Allergic Rhinitis Symptoms in the Past 12 Months (Current Rhinitis)		Adolescent’s Allergic Rash Symptoms in the Past 12 Months (Current Eczema)	
	Crude OR (95% CI) ^§^	Adjusted ^†^ OR (95% CI)	Crude OR (95% CI)	Adjusted OR (95% CI)	Crude OR (95% CI)	Adjusted OR (95% CI)
**Parental Education level: Primary/Secondary (** ***n*** **= 654)**						
Adherence to an active physical activity lifestyle every week ^‡^ (Yes vs. No)	0.24 (0.14–0.39) *	**0.56 (0.34–0.98)**	1.37 (0.82–2.28)	1.59 (0.95–2.67)	**0.41 (0.43–0.87)**	**0.62 (0.44–0.89)**
High consumption (most or all days) of fruits, vegetables, and pulses per week (Yes vs. No)	**0.44 (0.17–0.95)**	**0.42 (0.29–0.92)**	0.53 (0.37–1.21)	0.57 (0.46–1.24)	**0.34 (0.12–0.76)**	**0.52 (0.32–0.78)**
High consumption (most or all days) of starchy products (cereal, bread, pasta, and rice) per week (Yes vs. No)	0.24 (0.05–1.07)	0.39 (0.09–1.73)	1.09 (0.54–2.21)	1.21 (0.59–2.45)	0.63 (0.22–1.77)	1.08 (0.39–2.95)
High consumption (most or all days) of dairy (milk, yogurt, and cheese) per week (Yes vs. No)	**0.23 (0.14–0.37)**	**0.45 (0.26–0.79)**	**0.57 (0.43–0.77)**	**0.71 (0.51–0.98)**	**0.33 (0.22–0.51)**	0.85 (0.51–1.43)
Low consumption (up to twice) of unhealthy foods (fast-food, sweets, lollies, and soft drinks) per week (Yes vs. No)	**0.18 (0.12–0.29)**	**0.38 (0.22–0.64)**	**0.44 (0.33–0.58)**	**0.54 (0.39–0.76)**	**0.16 (0.10–0.24)**	**0.39 (0.23–0.66)**
**Parental Education level: Tertiary (*n* = 1280)**						
Adherence to an active physical activity lifestyle every week ^‡^ (Yes vs. No)	**0.12 (0.03–0.50)**	**0.18 (0.04–0.78)**	0.86 (0.65–1.13)	1.02 (0.77–1.36)	**0.27 (0.17–0.41)**	**0.39 (0.22–0.78)**
High consumption (most or all days) of fruits, vegetables, and pulses per week (Yes vs. No)	**0.14 (0.11–0.37)**	**0.17 (0.12–0.40)**	**0.17 (0.07–0.39)**	**0.19 (0.08–0.45)**	**0.17 (0.08–0.35)**	**0.35 (0.16–0.75)**
High consumption (most or all days) of starchy products (cereal, bread, pasta, and rice) per week (Yes vs. No)	0.48 (0.19–1.21)	1.92 (0.77–4.82)	0.84 (0.44–1.60)	1.19 (0.62–2.28)	0.39 (0.16–1.03)	1.03 (0.42–2.54)
High consumption (most or all days) of dairy (milk, yogurt, and cheese) per week (Yes vs. No)	**0.14 (0.09–0.20)**	**0.22 (0.17–0.45)**	**0.47 (0.38–0.59)**	**0.31 (0.18–0.49)**	**0.28 (0.21–0.38)**	0.74 (0.52–1.05)
Low consumption (up to twice) of unhealthy foods (fast-food, sweets, lollies, and soft drinks) per week (Yes vs. No)	**0.15 (0.11–0.21)**	**0.59 (0.38–0.87)**	**0.40 (0.33–0.50)**	**0.36 (0.23–0.52)**	**0.42 (0.36–0.59)**	**0.22 (0.05–0.92)**

^†^ Adjusted for sex, obesity status, parental atopic history, pet ownership, parental smoking, having an older sibling, exposure to indoor dampness and/or mold ^‡^ engaging in vigorous physical activity for >3 h/day plus watching TV and engaging in computer and internet activities for less than 3 h per day (*n*, %) ^§^ OR (95% CI): Odds Ratio (95% Confidence Interval) * Bold numbers denote *p* < 0.05.

## Data Availability

Upon reasonable request.
